# Relationship between Body Mass Index and Mortality in HIV-Infected HAART Users in the Women's Interagency HIV Study

**DOI:** 10.1371/journal.pone.0143740

**Published:** 2015-12-23

**Authors:** Anjali Sharma, Donald R. Hoover, Qiuhu Shi, Deborah Gustafson, Michael W. Plankey, Ronald C. Hershow, Phyllis C. Tien, Elizabeth T. Golub, Kathryn Anastos

**Affiliations:** 1 Department of Medicine, Albert Einstein College of Medicine, Bronx, New York, United States of America; 2 Department of Statistics and Biostatistics, Rutgers University, Piscataway, New Jersey, United States of America; 3 Department of Epidemiology and Community Health, New York Medical College, Valhalla, New York, United States of America; 4 Department of Neurology, State University of New York Downstate Medical Center, Brooklyn, New York, United States of America; 5 Department of Medicine, Division of Infectious Diseases, Georgetown University Medical Center, Washington, District of Columbia, United States of America; 6 Department of Epidemiology, University of Illinois at Chicago, Chicago, Illinois, United States of America; 7 Department of Medicine, Division of Infectious Diseases, University of California San Francisco and San Francisco Veterans Affairs Medical Center, San Francisco, California, United States of America; 8 Department of Epidemiology, Johns Hopkins Bloomberg School of Public Health, Baltimore, Maryland, United States of America; FIOCRUZ, BRAZIL

## Abstract

**Background:**

Early HIV studies suggested protective associations of overweight against mortality, yet data are lacking for the era of potent highly active antiretroviral therapy (HAART). We evaluated associations of pre-HAART initiation body mass index (BMI) with mortality among HAART-using women.

**Methods:**

Prospective study of time to death after HAART initiation among continuous HAART users in the Women’s Interagency HIV Study. Unadjusted Kaplan–Meier and adjusted proportional hazards survival models assessed time to AIDS and non-AIDS death by last measured pre-HAART BMI.

**Results:**

Of 1428 continuous HAART users 39 (2.7%) were underweight, 521 (36.5%) normal weight, 441 (30.9%) overweight, and 427 (29.9%) obese at time of HAART initiation. A total of 322 deaths occurred during median follow-up of 10.4 years (IQR 5.9–14.6). Censoring at non-AIDS death, the highest rate of AIDS death was observed among underweight women (p = 0.0003 for all 4 categories). In multivariate models, women underweight prior to HAART died from AIDS more than twice as rapidly vs. normal weight women (aHR 2.04, 95% CI 1.03, 4.04); but being overweight or obese (vs. normal weight) was not independently associated with AIDS death. Cumulative incidence of non-AIDS death was similar across all pre-HAART BMI categories.

**Conclusions:**

Among continuous HAART-using women, being overweight prior to initiation was not associated with lower risk of AIDS or non-AIDS death. Being underweight prior to HAART was associated with over double the rate of AIDS death in adjusted analyses. Although overweight and obesity may be associated with many adverse health conditions, neither was predictive of mortality among the HAART-using women.

## Introduction

Wasting, commonly experienced by people living with HIV early in the epidemic, is now rare [1, 2] whereas obesity and overweight are common nutritional problems for HIV-infected adults in the United States. Current prevalence estimates of overweight and obesity in HIV-infected women range from 58–78%, and are similar in women without HIV infection [3–6]. In general, being overweight and obese are associated with hypertension, dyslipidemia, cardiovascular disease, and insulin resistance; conditions now common among HIV-infected persons and expected to become more prevalent with aging [7, 8].

Among HIV-infected persons, several early studies suggested that being overweight or obese was associated with better, not poorer, prognoses and immunologic profiles and this perception remains today among HIV-infected persons. A report from the Women's Interagency HIV Study (WIHS) indicated that being overweight, obese, or morbidly obese was independently associated with higher CD4+ count, total lymphocyte count, and WBC counts compared to those of normal body mass index (BMI) (18.5–24.9 kg/m^2^) women. Morbid obesity (BMI ≥ 40 kg/m^2^) was associated with higher CD8+ counts [9]. Studies of mortality risk conducted among HIV-infected populations even earlier in the HIV epidemic suggested protective associations of overweight against mortality [10–12]; it is unclear whether this extends into the current era of potent antiretroviral therapy. Although studies of obesity and immunologic outcomes in HIV-negative populations have reported mixed results, this protective association of overweight against mortality exists among older HIV-uninfected adults. A recent systematic review and meta-analysis found that in the general U.S. population, overweight was associated with lower all-cause mortality compared with normal weight, whereas obesity and morbid obesity were associated with higher all-cause mortality [13]. We undertook this study to assess the association between BMI and risk of AIDS-specific and non-AIDS mortality among HIV-infected women regularly taking highly active antiretroviral therapy (HAART), which is currently recommended for all HIV-infected persons in the U.S. [14].

## Methods

### Study Population

The WIHS enrolled 2843 HIV-infected and 975 HIV-uninfected women in 1994–95 and 2001–02 from six sites (Bronx/Manhattan, Brooklyn, Chicago, Los Angeles, San Francisco and Washington DC). WIHS methods and baseline cohort characteristics have been described previously [15]. At semi-annual visits, participants have physical examinations and provide biological specimens and information on demographic and clinical characteristics, including HAART use. HIV-infected women in WIHS report use of all antiretroviral drugs during the previous six months at each study visit, with detailed questionnaires listing specific antiretroviral agents, and are prompted with the photo medication cards; interviewers also review participants’ medication lists and pill bottles when available. HAART is then defined via classification of the combined use of these antiretroviral agents based on published DHHS criteria [14]. The WIHS protocol was approved by the institutional review boards at each site, (Albert Einstein College of Medicine, Bronx, NY; State University of New York Downstate Medical Center; Brooklyn, NY; Rush University Medical Center and the Cook County Health and Hospital systems, Chicago IL; University of California, Los Angeles, CA; University of California, San Francisco, CA; Georgetown University Medical Center, Washington, District of Columbia; and Women’s Interagency HIV Study Data Management and Analysis Center, Johns Hopkins University, Baltimore MD), and all participants provided written informed consent. This analysis included all WIHS participants who initiated HAART after WIHS enrollment, had at least one BMI measurement prior to HAART initiation, and reported HAART use at 70% or more of post-initiation visits (which is defined as continuous use of HAART) [16]. Individual women were allowed to have up to four consecutive missed visits or visits off HAART (i.e. in cases of otherwise long-term consistent use), although less than 5% of the total semiannual person-visits were off HAART [16]. The HAART initiation date was the mid-date between the last pre-HAART, and the first post-HAART visit dates.

### Exposure Variables

The primary exposure of interest was BMI immediately (~ within 6 months) prior to initiation of HAART, which was determined by dividing body weight (kg) by height^2^ (m^2^) and categorized using standard BMI categories developed by the World Health Organization and National Heart, Lung, and Blood Institute as: underweight (<18.5), normal weight (18.5 to <25.0), overweight (25.0 to <30.0), and obese (≥30.0) [17, 18]. Use of predefined, standard BMI categories has been noted to avoid issues of ad hoc and post hoc selection of categories, and allows for comparison of results across studies [19, 20]. Other variables examined included calendar year of HAART initiation; age at HAART initiation; time of WIHS enrollment (1994–95 or 2001–02); HIV acquisition risk [injection drug use (IDU), heterosexual exposure, transfusion/other]; self-reported race: white (Hispanic and non-Hispanic), black (Hispanic and non-Hispanic) and other (predominantly women who self-identified as Hispanic but not white or black); and pre-HAART initiation variables of: self reported current illicit drug use (cocaine, crack, or heroin); alcohol use: none, light (<3 drinks/wk), moderate (3–13 drinks/wk), or heavy (≥14 drinks/wk); smoking tobacco (current, past, or never); annual income of $12,000 or less; having graduated from high school; depressive symptom burden defined as Center for Epidemiology Studies Depression score (CES-D) of ≥16 [21]; self-reported pre-HAART initiation AIDS defining illness (ADI); Hepatitis C Virus (HCV) infection defined as positive antibody with viremia; diabetes mellitus as previously used in WIHS [22]; CD4+ cell count measured at last visit preceding HAART initiation (within 12 months prior) was fit both as a continuous variable and categorized (as <200 cells/mm^3^, 200–350, or >350); and nadir CD4+ cell count (defined as the lowest CD4+ cell count measured at any visit prior to HAART); last pre-HAART log_10_ viral load (within 12 months prior to HAART initiation) and peak log_10_ HIV-1 RNA viral load (greatest HIV RNA level measured at any pre-HAART visit). We adjusted for WIHS site in all analyses.

### Outcome Variables

Primary outcomes were times from date of first visit after HAART initiation (index visit) to AIDS death, non-AIDS death, and death from all causes. The first visit after HAART initiation was chosen as the start point since a minimum of two visits with reported HAART use was needed to qualify as a continuous HAART user. Ascertainment and classification of deaths in WIHS have been described previously [23, 24]. ADIs were self-reported and classified as incident as previously described, based on the Centers for Disease Control expanded AIDS surveillance case definition [25]. Briefly, cause of death was determined from death certificates and electronic death certificate information from the National Death Index, with immediate and underlying causes of death, using the International Classification of Diseases, Ninth Revision (ICD-9), as well as local death registries, hospital records, physician reports, and reports from family or friends. Deaths were classified as due to unknown causes if there were no available death certificates, matches in the National Death Index, or clinical reports. Two physician-investigators reviewed the classification of each death.

Deaths were attributed to AIDS if the stated cause of death was an AIDS-defining opportunistic infection or malignancy; the stated cause of death was non-specific infection or organ failure (such as pneumonia with no organism identified, sepsis, or respiratory failure) with last CD4 count obtained <200 cells/mm^3^; or the stated cause of death was listed as AIDS and last CD4 count obtained was <200 cells/mm^3^.

Death was categorized as a non-AIDS death if a non-AIDS cause was identified as the primary cause of death (e.g., violence, accident, drug overdose, liver failure, non-AIDS–associated malignancy); or if kidney failure, or cardiovascular, gastrointestinal, or central nervous system disease was the primary cause of death and the last CD4 count was ≥200 cells/mm3.

Cause of death was considered to be indeterminate if the available cause of death was nonspecific (i.e. “cardiopulmonary arrest”); or listed cause of death was nonspecific infection or organ failure with the last CD4 count ≥200 cells/mm^3^; the stated cause was AIDS alone and the last CD4 count was ≥200 cells/mm^3^; or the primary cause of death was kidney failure with a CD4 count <200 cells/mm^3^. To account for loss to follow-up, women without study outcomes were censored at the earlier of either: (1) two years after their last HAART use visit or (2) December 31, 2010, as ascertainment of death beyond that date may not have been complete.

### Laboratory Methods

Plasma HIV-1 RNA was measured by isothermal nucleic acid sequence-based amplification (NASBA/Nuclisens; Organon Teknika Corp., Durham, NC, USA) with a lower limit of detection (LLD) of 80 copies/ml until October 1, 2008 and then by COBAS Taqman HIV-1 assay with LLD of 48 or 20 copies/mL. Lymphocyte subsets were quantified using standard flow cytometric methods in laboratories participating in the NIH/NIAID Flow Cytometry Quality Assessment Program [26]. HCV RNA was measured on frozen specimens from HCV antibody-positive women using either the COBAS Amplicor Monitor 2.0 assay or the COBAS Taqman assay (Roche Diagnostics, Branchburg, NJ, USA) [27].

### Statistical Analyses

Difference in means and proportions of the potential confounding covariates across BMI categories were determined by exact and rank tests. We performed Kaplan–Meier and Cox proportional hazards models of times to AIDS death and to non-AIDS death by BMI group [28]. For models of time to AIDS death we censored at non-AIDS death and vice-versa which returns a “cumulative incidence” that reflects an absolute probability of death if each cause of death were independent of the other. Multivariate proportional hazards analyses were performed with stepwise selection using a p-value of 0.1, with the following covariates assessed at the pre-HAART visit: race, age, HIV exposure category, alcohol consumption, income, education, date of WIHS enrollment, year of HAART initiation, current illicit drug use, depressive symptoms, last pre-HAART CD4+ count and category, nadir pre-HAART CD4+ count, last pre-HAART HIV-1 RNA viral load, peak HIV-1 RNA viral load, pre-HAART ADI, diabetes mellitus, and HCV status. As the primary exposure of interest, BMI was forced into the stepwise models, as was study site. To address potential confounding by illness-induced weight loss, sensitivity analyses were conducted by excluding those who died within the first 2 years of follow-up.

## Results

### Participant Characteristics

Between June 26, 1995 to April 2, 2013, 1428 participants met the criteria for continuous HAART use. Among these participants, the median BMI was 26.5kg/m^2^ with an interquartile range of 23.0–31.1 kg/m^2^; 39 (2.7%) were underweight (BMI <18.5), 521 (36.5%) normal weight (18.5 <BMI ≤ 25), 441 (30.9%) overweight (25 <BMI ≤ 30), and 427 (29.9%) obese (BMI ≥ 30)prior to HAART initiation (Table 1) A total of 322 deaths occurred during median follow-up of 10.4 years (IQR 5.9–14.6). Participant characteristics are shown in Table 1. Women with lower pre-HAART BMI category were more likely to report current illicit drug use, smoke cigarettes, and have HCV, but were less likely to have diabetes mellitus than those with higher BMI. Women with lower BMI were more likely to have markers of advanced HIV disease, including prior ADI, lower CD4+ nadir, and higher peak HIV RNA. Only 56% of women who were underweight pre-HAART were alive at the end of the study period, compared to 75% of those normal weight, 78% of those overweight, and 82% of those obese. A total of 167 AIDS and 143 non-AIDS deaths occurred during follow-up. Leading causes of non-AIDS deaths included malignancy in 27%, liver disease in 17%, heart disease in 17%, drug/alcohol overdose in 14%, and neurologic causes in 4%. Of the 143 women who progressed to non-AIDS-related deaths, 39 were overweight and 35 were obese. Among these non-AIDS deaths, we classified heart disease, malignancy, and neurological causes as potentially obesity-related. These diagnoses accounted for 54% of non-AIDS deaths among obese women, compared to 46% of deaths in normal and overweight women.

**Table 1:** Participant Characteristics.

	Underweight (BMI< 18.5) N = 39	Normal (18.5 <BMI ≤ 25) N = 521	Overweight (25 <BMI ≤ 30, N = 441	Obese (BMI≥30) N = 427	P Value
**Age at last Pre-HAART visit (Yrs)**	40.2 ± 8.0	40.6 ± 8.6	40.2 ± 8.1	40.9 ± 8.3	0.64
**Baseline risk category**					0.34
** Injection Drug Use**	13 (33.3%)	146 (28.3%)	130 (29.8%)	104 (24.6%)	
** Heterosexual risk**	17 (43.6%)	235 (45.5%)	168 (38.5%)	183 (43.3%)	
** Transfusion risk**	1 (2.6%)	14 (2.7%)	13 (3.0%)	16 (3.8%)	
** Unknown/other**	8 (20.5%)	121 (23.5%)	125 (28.7%)	120 (28.4%)	
**Race**					0.0002
** White**	8 (20.5%)	147 (28.3%)	99 (22.5%)	67 (15.7%)	
** Black**	26 (66.7%)	258 (49.6%)	247 (56.0%)	269 (63.0%)	
** Other**	5 (12.8%)	115 (22.1%)	95 (21.5%)	91 (21.3%)	
**Baseline annual income ≤ $12,000**	24 (66.7%)	256 (52.1%)	249 (59.4%)	222 (55.0%)	0.08
**Baseline education level (% completing high school or more)**	27 (69.2%)	348 (67.1%)	252 (57.1%)	278 (65.1%)	0.009
**WIHS Study Site**					0.06
** Bronx**	6 (15.4%)	88 (16.9%)	83 (18.8%)	94 (22.0%)	
** Brooklyn**	4 (10.3%)	75 (14.4%)	66 (15.0%)	84 (19.7%)	
** Washington D.C.**	4 (10.3%)	71 (13.6%)	61 (13.8%)	62 (14.5%)	
** Los Angeles**	8 (20.5%)	112 (21.5%)	99 (22.5%)	81 (19.0%)	
** San Francisco**	12 (30.8%)	89 (17.1%)	64 (14.5%)	61 (14.3%)	
** Chicago**	5 (12.8%)	86 (16.5%)	68 (15.4%)	45 (10.5%)	
**WIHS year of enrollment**					0.04
** 1995–95**	32 (82.1%)	428 (82.2%)	334 (75.7%)	323 (75.7%)	
** 2001–02**	7 (18.0%)	93 (17.9%)	107 (24.3%)	104 (24.4%)	
**Calendar year of HAART initiation**	2000.9 ± 4.3	2000.7 ± 4.2	2001.1 ± 4.1	2001.6 ± 4.5	0.01
**Current illicit drug use (cocaine, crack or heroin) at index visit**	9 (23.1%)	81 (15.6%)	47 (10.7%)	27 (6.3%)	< 0.0001
**Alcohol use**					0.004
** None**	25 (64.1%)	272 (52.3%)	276 (62.9%)	264 (62.0%)	
** Light**	8 (20.5%)	150 (28.9%)	110 (25.1%)	117 (27.5%)	
** Moderate**	5 (12.8%)	70 (13.5%)	41 (9.3%)	38 (8.9%)	
** Heavy**	1 (2.6%)	28 (5.4%)	12 (2.7%)	7 (1.6%)	
**Smoking status**					0.001
** Never**	7 (18.0%)	145 (27.8%)	125 (28.3%)	141 (33.0%)	
** Past**	5 (12.8%)	110 (21.1%)	110 (24.9%)	119 (27.9%)	
** Current**	27 (69.2%)	266 (51.1%)	206 (46.7%)	167 (39.1%)	
**Depressive symptoms, CESD> = 16 (%)**	21 (53.9%)	224 (43.4%)	195 (44.8%)	193 (45.6%)	0.61
**Nadir Pre-HAART CD4+ count (cells/mm** ^**3**^ **)**	157 ± 117	192 ± 135	215 ± 157	243 ± 175	< 0.0001
**Last Pre-HAART CD4+ count (cells/mm** ^**3**^ **) category**					<0.0001
** < 200**	16 (42.1%)	170 (32.7%)	128 (29.2%)	92 (21.6%)	
** 200–350**	11 (29.0%)	171 (32.9%)	124 (28.3%)	126 (39.6%)	
** > 350**	11 (29.0%)	179 (34.4%)	186 (42.5%)	208 (48.8%)	
**Peak log**_**10**_ **HIV RNA viral load**	4.98 ± 0.87	4.81 ± 0.82	4.76 ± 0.80	4.65 ± 0.86	0.006
**Last Pre-HAART log**_**10**_ **HIV RNA viral load**	3.47 ± 1.38	3.05 ± 1.25	2.97 ± 1.20	2.96 ± 1.17	0.06
**Prior AIDS-defining illness**	25 (64.1%)	282 (54.1%)	208 (47.2%)	191 (44.7%)	0.006
**Hepatitis C viremia**	14 (35.9%)	160 (30.7%)	135 (30.6%)	101 (23.7%)	0.04
**Diabetes Mellitus**	0	16 (3.07%)	30 (6.80%)	57 (13.4%)	< 0.0001
**Vital status at end of study**					0.009
** Alive**	22 (56.4%)	390 (74.9%)	343 (77.8%)	351 (82.2%)	
** AIDS Death**	11 (28.2%)	63 (12.1%)	55 (12.5%)	38 (8.9%)	
** Non-AIDS Death**	6 (15.4%)	63 (12.1%)	39 (8.8%)	35 (8.2%)	
** Unknown/indeterminate**	0	5 (1.0%)	4 (0.9%)	3 (0.7%)	

All predictors are pre-HAART unless specified

### Cumulative Incidence of AIDS and non-AIDS Death

**Fig 1:**
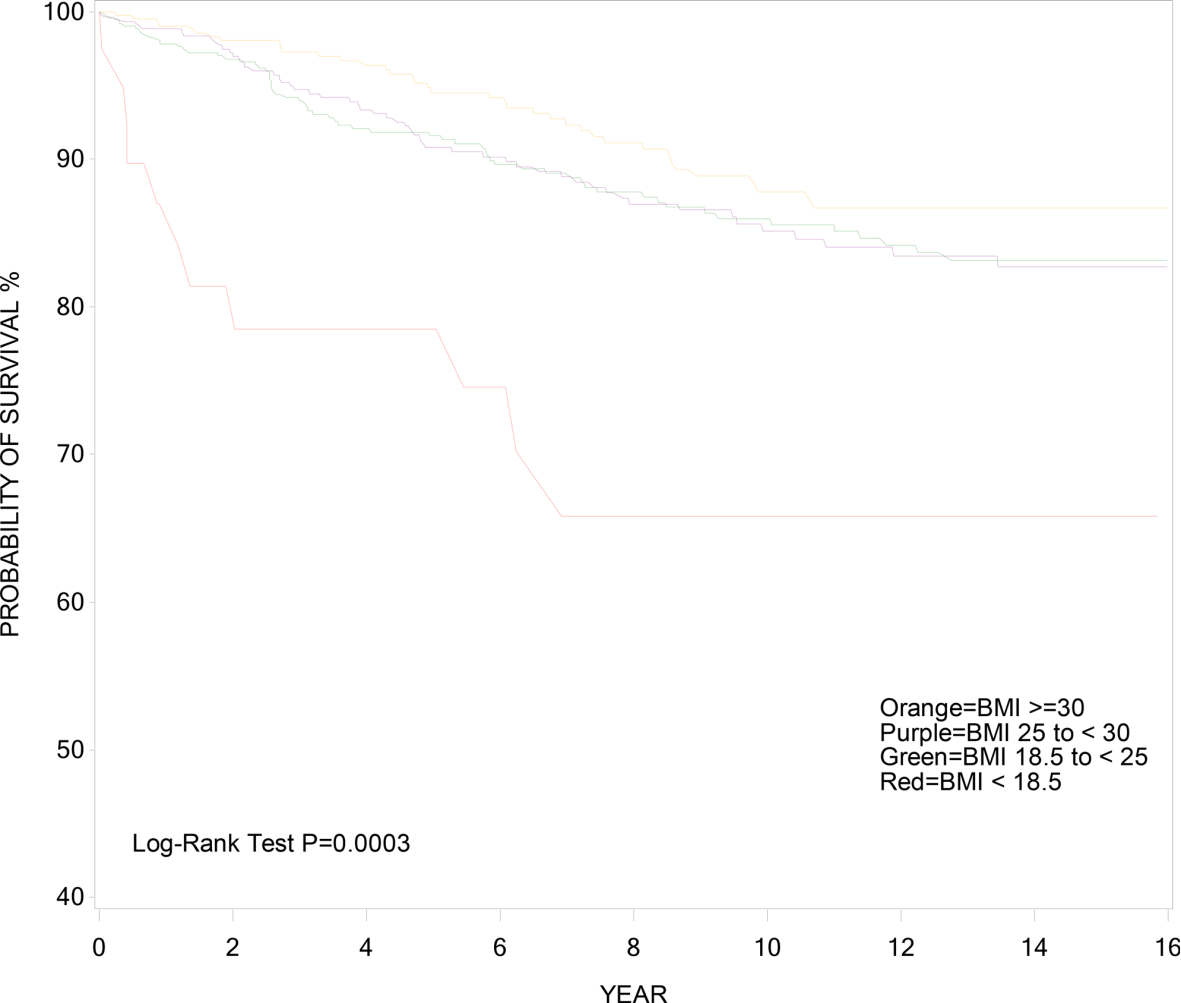
Cumulative Incidence of AIDS Survival.

**Fig 2:**
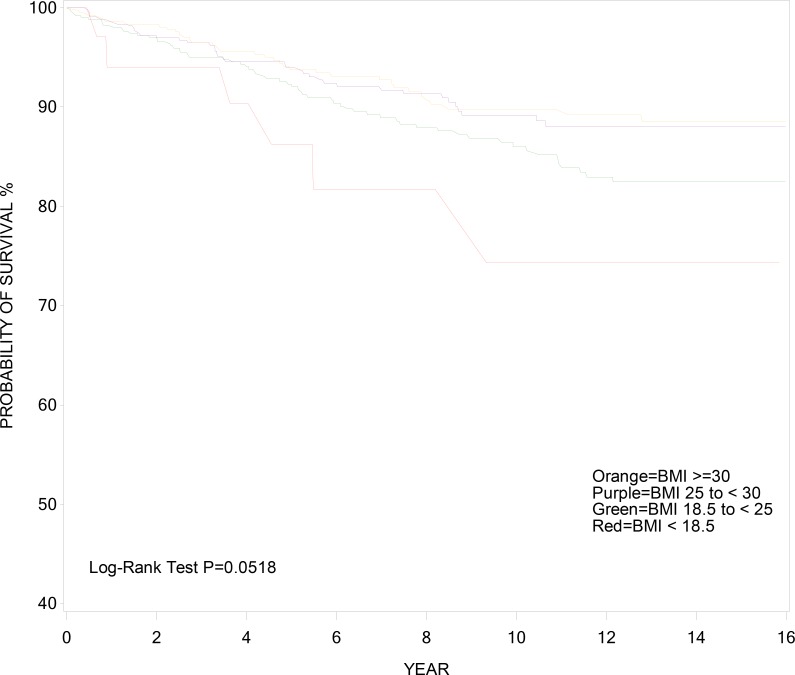
Cumulative Incidence of Non-AIDS Survival.

Figs 1 and 2 show the cumulative incidence of AIDS death (with censoring by non-AIDS death) and non-AIDS death (with censoring at AIDS death) over time, respectively, stratified by pre-HAART BMI. Cumulative incidence of AIDS and non-AIDS death in these figures reflect what survival would be if occurrence of each type of death was independent of the other. Women who were underweight prior to HAART use had the highest incidence of AIDS death at 2-year and 10-year after HAART initiation (18.6% and 34.2%) when compared with other BMI categories, (3.2% and 14.0%) for normal weight, (3.1% and 14.9%) for overweight, and (1.9% and 12.2%) for obese (log-rank p = 0.0003) (Fig 1). Incidence of non-AIDS death was similar across BMI categories, (6.0% and 25.7%) at 2-year and 10-year post-HAART initiation for underweight, (3.0% and 13.9%) for normal weight, (3.0% and 10.8%) for overweight, and (1.7% and 10.2%) for obese (log-rank p = 0.05) (Fig 2).

### Associations with Time to AIDS Deaths

In univariate and multivariate Cox proportional analyses, underweight women died more rapidly from AIDS than did women in all other weight categories (Table 2) In multivariate models, continuous HAART users who were underweight pre-HAART died from AIDS more than twice as rapidly as normal weight women (adjusted hazard ratio (aHR) 2.04, 95% CI 1.03, 4.04; p = 0.042). Being overweight, obese, or morbidly obese was not associated with hazard of AIDS death. Other predictors of AIDS death included black race (aHR 1.87, 95% CI 1.14, 3.05; p = 0.01; ref: white), depressive symptom burden (aHR 2.17, 95% CI 1.56, 3.00; p<0.0001), and HCV infection (aHR 1.54, 95% CI 1.10, 2.15; p = 0.011), whereas later year of HAART initiation was associated with a lower hazard of AIDS death (aHR 0.92 per year, 95% CI 0.86, 0.97; p = 0.005). Markers of HIV disease severity were independently predictive of AIDS death, including last pre-HAART CD4+ count below 200 cells/mm^3^, prior ADI, and both last pre-HAART log_10_ HIV RNA viral load r and peak log_10_ HIV RNA viral load (Table 2). Exclusion of early deaths (within 2 years of HAART initiation) did not significantly change the results.

**Table 2:** Predictors of AIDS Death.

	Univariate Model		Multivariate Model	
	HR (95% CI)	P Value	aHR (95% CI)	P Value
**BMI category**				
** Underweight (BMI of <18.5)**	3.05 (1.60, 5.80)	0.0007	2.04 (1.03, 4.04)	0.042
** Normal weight (BMI of 18.5-<25)**	**Reference**			
** Overweight (BMI of 25-<30)**	0.99 (0.69, 1.42)	0.96	1.03 (0.71, 1.9)	0.87
** Obese (BMI of ≥30)**	0.71 (0.47, 1.06)	0.09	0.91 (0.60, 1.39)	0.67
**Race**				
** Black vs. White**	2.23 (1.38, 3.60)	0.001	1.87 (1.14, 3.05)	0.01
** Other vs. White**	1.69 (0.97, 2.92)	0.06	1.64 (0.94, 2.90)	0.08
**Age at Visit, per 5 yr**	1.06 (0.97, 1.17)	0.20		
**Baseline annual income ≤ $12,000**	1.64 (1.18, 2.29)	0.004		
**Calendar year of HAART initiation**	0.87 (0.82, 0.92)	<0.0001	0.92 (0.86, 0.97)	0.005
**Smoking status**				
** Former smoker vs. never smoker**	0.82 (0.50, 1.35)	0.44		
** Current smoker vs. never smoker**	1.65 (1.13, 2.42)	0.009		
**CES-D 16+ vs. <16**	2.39 (1.74, 3.28)	<0.0001	2.17 (1.56, 3.00)	<0.0001
**Nadir Pre-HAART CD4+ count (per 100 c/mm** ^**3**^ **)**	0.55 (0.48, 0.64)	<0.0001		
**Last Pre-HAART CD4+ count (cells/mm** ^**3**^ **)**				
** CD4 < 200 vs. > 350**	5.46 (3.62, 8.25)	<0.0001	3.05 (1.96, 4.76)	<0.0001
** CD4 200–350 vs. > 350**	1.72 (1.07, 2.78)	0.03	1.47 (0.90, 2.39)	0.12
**Prior AIDS-defining illness**	2.70 (1.95, 3.97)	<0.0001	1.51 (1.06, 2.17)	0.02
**Last pre-HAART log**_**10**_ **HIV RNA viral load**	1.87 (1.64, 2.12)	<0.0001	1.32 (1.14, 1.52)	0.0002
**Peak log**_**10**_ **HIV RNA viral load**	2.38 (1.91, 2.97)	<0.0001	1.48 (1.15, 1.90)	0.002
**Hepatitis C Viremia**	2.07 (1.51, 2.83)	<0.0001	1.54 (1.10, 2.15)	0.011

Outcomes shown are hazard ratios (HR) or adjusted hazard ratios (aHR); all predictors are pre-HAART unless specified; BMI: Body Mass Index (calculated as weight in kilograms divided by height in meters squared); CES-D: Center for Epidemiologic Studies Depression Scale; CI, confidence interval; Multivariate models were created using stepwise proportional hazards with BMI category forced in, and adjusted for Women's Interagency HIV Study site.

### Associations with Time to non-AIDS Deaths

In univariate and multivariate Cox proportional models, pre-HAART BMI was not statistically associated with non-AIDS death in continuous HAART users (Table 3) Overweight and obese BMI categories were associated with a non-significant reduction in the hazard of non-AIDS death compared to normal weight women (aHR 0.68, 95% CI 0.45, 1.02; p = 0.06, and aHR 0.72, 95% CI 0.47, 1.12; p = 0.14 respectively). Age (aHR 1.21 per 5 years, 95% CI 1.08, 1.35; p = 0.001), annual income of $12,000 or less (aHR 1.67, 95% CI 1.12, 2.48; p = 0.01), current cigarette smoking compared with never smoking (aHR 2.25, 95% CI 1.31, 3.89; p = 0.0035), diabetes mellitus (aHR 2.20, 95% CI 1.28, 3.79; p = 0.005), and HCV (aHR 1.82, 95% CI 1.25, 2.65; p = 0.002), were associated with greater hazard of non-AIDS death. Later year of HAART initiation was associated with reduced hazard of non-AIDS death (aHR 0.93 per year, 95% CI 0.88, 0.98; p = 0.01). Sensitivity analyses performed excluding diabetes mellitus or early deaths from the models did not substantially change the hazards of non-AIDS death for any BMI category, nor any of the other factors listed above.

**Table 3:** Predictors of Non-AIDS Death.

	Univariate Model		Multivariate Model	
	HR (95% CI)	P Value	aHR (95% CI)	P Value
**BMI category**				
** Underweight (BMI of <18.5)**	1.56 (0.67, 3.62)	0.30	1.11 (0.44, 2.81)	0.82
** Normal weight (BMI of 18.5-<25)**	Reference		Reference	
** Overweight (BMI of 25-<30)**	0.71 (0.48, 1.07)	0.10	0.68 (0.45, 1.02)	0.06
** Obese (BMI of ≥30)**	0.72 (0.48, 1.10)	0.13	0.72 (0.47, 1.12)	0.14
**Age at Visit, per 5 yr**	1.25 (1.14, 1.37)	<0.0001	1.21 (1.08, 1.36)	0.001
**Baseline annual income ≤ $12,000**	2.24 (1.53, 3.27)	<0.0001	1.67 (1.12, 2.48)	0.01
**Calendar year of HAART initiation**	0.95 (0.91, 1.01)	0.08	0.93 (0.88, 0.98)	0.01
**Smoking status**				
** Never smoker**	Reference		Reference	
** Former smoker**	1.25 (0.68, 2.28)	0.47	1.38 (0.74, 2.57)	0.32
** Current smoker**	2.92 (1.79, 4.78)	<0.0001	2.25 (1.31, 3.89)	0.004
**Diabetes Mellitus**	2.23 (1.34, 3.71)	0.002	2.20 (1.28, 3.79)	0.005
**Hepatitis C Viremia**	3.00 (2.13, 4.21)	<0.0001	1.82 (1.25, 2.65)	0.002

Outcomes shown are hazard ratios (HR) or adjusted hazard ratios (aHR); all predictors are pre-HAART unlessspecified; BMI: Body Mass Index (calculated as weight in kilograms divided by height in meters squared); CI, confidence interval; Models were created using stepwise proportional hazards with BMI category forced in, and adjusted for Women’s Interagency HIV Study site.

### Associations with Time to All-Cause Death

In univariate Cox proportional analyses of all-cause mortality, underweight women died more rapidly than did women with normal BMI category (HR 2.22, 95% CI 1.33, 3.69; p = 0.002), whereas obesity was associated in a reduction in mortality (HR 0.71, 95% CI 0.53, 0.94; p = 0.018). In multivariate models, neither underweight BMI (aHR 1.66, 95% CI 0.94, 2.91; p = 0.079) nor obesity (aHR 0.91, 95% CI 0.67, 1.23; p = 0.55) was significantly associated with all-cause mortality among continuous HAART users. Overweight was not associated with all-cause mortality in unvariate models (HR 0.85, 95% CI 0.66, 1.11; = 0.23) or in multivariate analyses (aHR 0.87, 95% CI 0.66, 1.14; p = 0.31). Factors associated with increased all-cause mortality in multivariate models included age (aHR 1.14, 95% CI 1.05, 1.23; p = 0.002), annual income of $12,000 or less (aHR 1.33, 95% CI 1.03, 1.72; p = 0.029), current smoking (aHR 1.51, 95% CI 1.09, 2.10; p = 0.01),), depressive symptom burden (aHR 1.58, 95%CI 1.24, 2.01; p = 0.0002), diabetes mellitus (aHR 1.71, 95% CI 1.14, 2.56; p = 0.009), and HCV infection (aHR 1.52, 95% CI 1.18, 1.98; p = 0.002), in addition to pre-HAART and peak HIV RNA viral load, and pre-HAART CD4+ count below 200cells/mm^3^ (aHR 2.41, 95% CI 1.78, 3.26; p<0.0001).

## Discussion

We found that overweight and obesity were neither associated with an increased risk of non-AIDS mortality nor were they protective of AIDS mortality among women regularly receiving long-term HAART. The directionality of the hazard ratios suggests that higher BMI may have a protective association with non-AIDS death, and it is possible that as this cohort of middle-aged women continues to grow older and more likely to develop conditions associated with both obesity and increased risk of mortality, the relationships between overweight, obesity, and mortality in HIV-infected women may become more apparent.

A number of studies conducted in the general U.S. population have shown associations between overweight and reduced mortality. In the National Health and Nutrition Examination Survey (NHANES) both underweight and obesity were associated with excess mortality, but overweight was associated with reduced mortality compared to normal weight [29]; and in a systematic review, overweight was associated with lower all-cause mortality, whereas obesity was associated with higher all-cause mortality, both relative to normal weight [13]. Some studies have described a U-shaped relationship of BMI and mortality, with the lowest mortality risk seen near a BMI of 25 kg/m^2^ [30]. Yet the relationship of overweight to risk of death in the general U.S. population remains controversial. Moreover, the relationship between BMI and mortality varies by age [31, 32]. Some have suggested that the ideal weight for elderly people is greater than for younger people, while others postulate that the protective effect of weight observed in elderly people may represent selective survival, where obese people have died prematurely, or alternatively a cohort effect, reflecting temporal trends: older people come from cohorts with less obesity [32–34]. Thus, Given the mean age of 40 years prior to HAART use in this study, it is possible that a protective effect of overweight may manifest only in the WIHS cohort as these women approach older age.

Thresholds at which BMI confers mortality risk may also vary significantly by race. Several studies have reported a weaker association between higher BMI categories and mortality in black women than in white women, with thresholds for elevated mortality risk as high as BMI of ≥ 40 [35–42]; although the association between BMI and mortality was similar in black and white women in the Black Women’s Health Study and in the Multiethnic Cohort Study [43, 44]. The majority of participants in the WIHS cohort are black women, in whom the association between BMI and mortality has been reported to be weaker than in white women.

Among HIV-infected persons, studies of the relationship between overweight and obesity and immunologic and virologic outcomes conducted in the HAART era have shown conflicting results; however, these studies included only short-term follow-up and did not evaluate mortality endpoints. Several pre-HAART era studies have suggested that excess weight may actually be beneficial in HIV-infected populations in the U.S. In the Miami Intravenous Drug Abuse Study, among predominantly (91%) untreated HIV-infected drug users, obese subjects (defined as BMI ≥ 27 kg/m^2^) had decreased mortality risk compared to those nonobese (BMI <27 kg/m^2^) and underweight (BMI ≤ 19.5 in men and ≤ 18.5 in women), and a greater proportion of the non-obese group exhibited >25% CD4+ count decline over 18 months compared with obese participants [11]. Similarly, in an urban HIV clinic, Shuter reported an inverse relationship of both initial BMI and subsequent BMI with progression to AIDS [12]. In the HIV Epidemiology Research Study (HERS) cohort of HIV-infected women, higher baseline BMI was associated with a lower rate of occurrence of the first CD4+ cell count < 200 cells/mm^3^; both underweight and normal weight women had an increased risk of progression to AIDS; and underweight women had an increased risk of AIDS-death compared with obese women [10].

We found that in continuous HAART-using women, being underweight prior to HAART carried over double the hazard of AIDS death than having normal weight, even after adjusting for confounders and measures of HIV disease severity. A number of studies in resource-limited settings have demonstrated that low BMI at the time of HAART initiation is predictive of early mortality in South East Asia [45, 46] and in sub-Saharan Africa [47–49], where malnutrition is prevalent. Prior to the widespread availability of antiretroviral therapy, studies in westernized settings also found relationships between low BMI in untreated HIV-infected individuals, and HIV disease progression and death [50–53]. However wasting is now very infrequent in the United States, and the proportion of overweight and obese HIV-infected individuals has increased since the introduction of HAART [1]. In our study less than 3% of women were underweight, whereas the majority of women (61%) were overweight, obese, or morbidly obese, consistent with published prevalence estimates of overweight and obesity in HIV-infected women ranging from 58–78% in the U.S., and similar to that seen in U.S. women without HIV infection [3–6]. Our study is unique in that it is to date, the only long-term analysis conducted among continuous HAART users, which reflects standard of care for HIV infection in the U.S.

Our study specifically looked at women continuously taking HAART over time, consistent with current treatment norms in the HAART era. Despite the established survival benefits of HAART, low pretreatment BMI remained an independent predictor of early AIDS mortality, which was limited to the first two years after HAART initiation. Our study extends the literature associating low BMI and mortality into the HAART era, demonstrating that these effects persist for the first two years after HAART initiation despite consistent use. Although AIDS deaths did occur after the initial 2 years following HAART initiation, the hazards of death did not appear to vary by initial pre-HAART BMI category beyond the first 2 years of continuous HAART use. The mechanisms placing underweight HIV-infected women on HAART at risk of AIDS death are unclear. Several factors which could have facilitated HIV disease progression and death were more prevalent among underweight study participants, including measures of advanced disease, HCV co-infection, and history of substance abuse; however this effect remained despite adjusting for measures of HIV disease severity such as prior AIDS defining illness, CD4 count and viral load, as well as HCV infection. While underweight women were more likely to be smokers and report depressive symptoms (and had lower CD4 nadir), these factors were not associated with AIDS death in multivariable analyses and did not account for the association between low BMI and AIDS death. Although some have reported improved immune benefits of being overweight such as greater CD4 response after HAART initiation [54], others have found smaller increases in CD4 counts and percentages in obese compared with normal weight persons, suggesting that obesity might impair CD4 recovery [55, 56]. Still others found no difference by weight in those achieving an undetectable viral load and CD4 increase at 3–9 months after HAART initiation, despite lower pretreatment viral load in obese persons [5]. It is possible that low BMI may be emblematic of underlying disease processes, placing underweight women on the pathway leading to AIDS death, which is not easily reversed despite HAART use.

We decided to use pre-HAART BMI rather than time-updated BMI or BMI trajectory in our analyses, because our objective was to determine the relationship of pre-HAART BMI to mortality, and specifically to determine the extent to which BMI prior to HAART initiation predicts mortality in HIV-infected continuous HAART users. Use of post-HAART BMI as a predictor of mortality as a time dependent variable also leads to the issue of reverse causality as many people lose weight shortly prior to death due to end stage consequences of the death process. Thus heavier weight would appear protective because impending death process is leading to reduced weight not that heavy weight in and of itself is being protective. Additionally, rather than model competing causes of death, we decided to stratify analyses based on whether or not cause of death was attributed to AIDS (vs. non-AIDS). We chose this approach because we hypothesized that the relationship between BMI and mortality might differ significantly in AIDS deaths vs. non-AIDS deaths. When we use AIDS death as an outcome then a non-AIDs death is a “competing risk” as death from a non-AIDs cause prevents a death from AIDS; and similarly when non-AIDS death is the outcome then AIDS death is a “competing risk”. We have no basis on which to model when a person who dies of an AIDS death would have died of a non-AIDS cause if they had not died of AIDS first; and similarly, we have no basis on how to model when a person who dies of an AIDs death would have died of a non-AIDS cause if they had not died of AIDS first. Thus, we believe that any attempt to fit such models would introduce more error and bias due to errors in the model assumptions.

Our study has several limitations. We did not have measures of energy balance, dietary intake, or physical activity. The WIHS is a middle-aged population and most Americans die at an older age than the women in WIHS; therefore we may not have been able to elucidate the mortality risk associated with excess body weight expected for non-AIDS death, despite the extended time period under study. Although the potentially protective association between overweight and non-AIDS death we observed was not statistically significant, we may have been underpowered to detect a significant association between overweight or obesity and mortality given the relatively few numbers of deaths observed in each BMI category. Accurately classifying obesity-related and non-obesity-related deaths was not possible because specific causes of death were often not identified. Although only certain cancers are obesity-related, even if we assume that all "cancer", "neurological", and "heart"-related deaths were obesity-related, they would account for only 69 of 143 (48%) non-AIDS deaths. Obese women who died from non-AIDS causes were slightly more likely to have died of potentially obesity-related outcomes than either overweight or normal weight women (54% for obese vs. 44% for overweight vs. 47% for normal weight). Although we evaluated and adjusted for markers of socioeconomic status, residual confounding is still possible. As in any observational study, unmeasured confounders may also affect these relationships.

In conclusion, we found that in continuous HAART-using women, having higher than normal BMI was not significantly associated with protection from AIDS or non-AIDS death. Being underweight (versus normal weight) prior to HAART increased the hazard of AIDS death two-fold in the first two years after HAART initiation, after adjusting for measures of HIV disease severity. Although overweight and obesity may be associated with a number of adverse health conditions, they conferred neither an increased or decreased risk of mortality among middle-aged HIV-infected women receiving long-term continuous HAART. Despite the survival benefits of potent antiretroviral therapy, women with low BMI are at risk for AIDS death for up to two years after HAART initiation.
